# Total Intravenous Anesthesia with Ketofol versus Combination of Ketofol and Lidocaine for Short-Term Anesthesia in Pediatric Patients; Double Blind, Randomized Clinical Trial of Effects on Recovery

**DOI:** 10.3390/children9020282

**Published:** 2022-02-18

**Authors:** Ana Nevešćanin Biliškov, Danijela Gulam, Marija Žaja, Zenon Pogorelić

**Affiliations:** 1 Department of Anesthesiology, Reanimatology and Intensive Care, University Hospital of Split, Spinčićeva 1, 21000 Split, Croatia; anevescanin@gmail.com (A.N.B.); marijazaja@hotmail.com (M.Ž.); 2 School of Medicine, University of Split, Šoltanska 2, 21000 Split, Croatia; danijela.gulam@gmail.com; 3 Department of Anesthesiology, Reanimatology and Intensive Care, General Hospital Šibenik, Stjepana Radića 83, 22000 Šibenik, Croatia; 4 Department of Pediatric Surgery, University Hospital of Split, Spinčićeva 1, 21000 Split, Croatia

**Keywords:** ketofol, lidocaine, TIVA, pediatric anesthesia, recovery

## Abstract

Background: Ketofol admixture has been proposed to be useful for sedation and general anesthesia. The beneficial effect of the combination of ketofol with lidocaine may be a shortened time of anesthesia and recovery period. This study aimed to establish the effect of total intravenous anesthesia (TIVA) with ketofol and ketofol with lidocaine on recovery in children. Methods: Two hundred children from the ages of 1–12 years who underwent short surgical procedures were randomly allocated into two groups. Propofol mixtures (ketofol) were prepared for group l. A ratio of 1:4 of ketofol was used for induction and for the maintenance of anesthesia a ratio of was used 1:7. For the induction and maintenance of anesthesia ketofol with lidocaine (lidoketofol) was used in group II. The McFarlan infusion regimen was used with reduction. The extubating time, anesthesia duration and the length of stay in the post-anesthesia care unit (PACU) were recorded. Results: Extubation time showed to be considerably shorter in the lidoketofol group than in the ketofol group (120 s versus 240 s; *p* < 0.00001). The anesthesia duration was also significantly shorter in the lidoketofol group (35 min vs. 50 min; *p* < 0.00001). The lidoketofol group showed to have a lower length of stay in the post-anesthesia care unit (PACU) than the ketofol group (20 min vs. 35 min; *p* < 0.00001). The lidoketofol group showed lower fentanyl consumption per kg (2.1 µg per kg vs. 2.3 µg per kg; *p* < 0.056) and lower propofol consumption (6.6 mg per kg vs. 7.6 mg per kg; *p* < 0.032). Conclusion: The recovery in pediatric anesthesia can improve with usage of TIVA with ketofol plus lidocaine admixture with a reduced McFarlan dose regimen.

## 1. Introduction

Pediatric anesthesia differs in many aspects from adult anesthesia because the needs of infants and young children are fundamentally different from those of adults. The pediatric anesthesiologist must understand the challenges and particularities of pediatric physiology [1]. The induction of anesthesia in children can be performed using inhalational and intravenous anesthetics nowadays, total intravenous anesthesia is an incredibly attractive choice for pediatric anesthesiologist. The increased trend of total intravenous anesthesia is due to its many advantages, such as a low incidence of postoperative vomiting, smooth recovery from anesthesia and improved quality of emergence from anesthesia [2,3].

Ketamine is an anesthetic drug with a wide range of effects including analgesic properties, cardiovascular stimulation resulting in increase in heart rate, arterial blood pressure and cardiac output. In addition, it produces minimal respiratory depression, thus a result of its minimal effects on central respiratory drive [4]. Propofol is an intravenous hypnotic drug with rapid recovery, differing in its effects from ketamine by decreasing the blood pressure and cardiac output. It results in respiratory depression by affecting the central chemoreceptor sensitivity [5]. The fusion of ketamine and propofol is referred to as ketofol. These two drugs are pharmacologically compatible, their combination produces sedation, improves analgesia and results in immediate recovery with hemodynamic stability and minimal respiratory depression [6]. In a previous study various ratios of ketofol have been described, the dose and ratio of the kefotol mixture determine its efficacy and safety [6,7]. Ketofol has been of growing interest over the recent years for sedation and analgesia in pediatric anesthesia [2].

Lidocaine is a local anesthetic of the amide group; it is the most often applied local anesthetic of its group. It exerts its effects by inactivating voltage-dependent sodium channels, thus altering signal conduction in neurons. Lidocaine can be used in various pain managements, as topical anesthesia, local, regional anesthesia and for various blocks [8]. The perioperative effects of lidocaine decrease inhalational anesthetic requirement, minimize the need for opioids, decrease postoperative nausea and vomiting and shorten the extubation time. In addition, perioperative effects of lidocaine are associated with a decrease in hospital stay [9,10]. Lidoketofol is a combination of ketofol and lidocaine. There are currently no studies with the mixture of ketofol and lidocaine used for induction and maintenance of anesthesia.

The purpose of the current study was to examine the effects of TIVA in a combination of ketofol and lidocaine and its effects on recovery from anesthesia. The manual infusion system for TIVA with lidoketofol is described in our current study. In addition, we looked into the effects of ketofol and lidoketofol infusion on recovery from anesthesia. We tested the hypothesis that TIVA with lidoketofol can provide improved recovery conditions in pediatric anesthesia.

## 2. Materials and Methods

### 2.1. Patients

**Figure 1:**
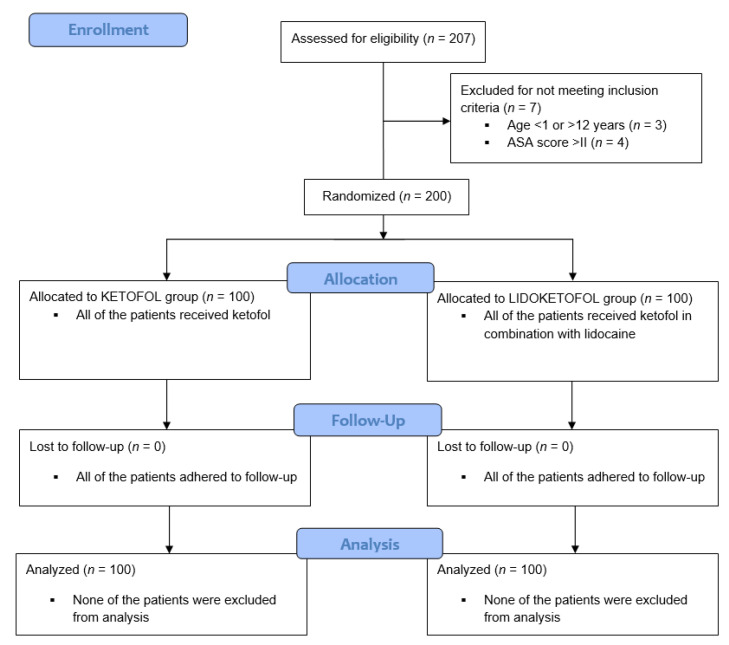
Flow chart of the study.

Two hundred pediatric patients who were scheduled for elective short-term surgery, were included in the study. Only the patients scheduled for elective procedures, with a duration less than 60 min in inguino-scrotal region (inguinal hernia repair, orchiopexy, orchiectomy, hydrocele/funiculocele repair, circumcision, hypospadias surgery) were included in study. The study has been conducted from June 2020 until December 2020 in the University hospital of Split, Croatia. Inclusion criteria were previously healthy children aged 1–12 years with an American Society of Anesthesiologists status (ASA) of I and II who underwent short-term surgery. Exclusion criteria were children outside the specified age range, children with ASA III-V, children with comorbidities and children with incomplete data during follow-up. Out of the patients that were included, 100 were in the ketofol group and 100 in the lidoketofol group. Legal representatives of all patients signed an informed consent. The flow chart diagram of the study is shown in Figure 1. Study protocol was approved by the Ethics Review Board of our hospital (reference number 2181-147-01/06/M.S.-20-12, Date of approval: 15 June 2020). Study was registered in ClinicalTrials.gov registry under identifier NCT04467424.

### 2.2. Intraoperative Monitoring

For standard monitoring non-invasive arterial blood pressure, electrocardiogram (ECG), and peripheral oxygen saturation (Draeger-Perseus A500 Anesthesia Device Monitor, Draeger Medical Systems, Inc., Denver, MA, USA) were used. The depth of anesthesia was monitored by bispectral index monitoring system (BIS; BIS™ Brain Monitoring System, Covidien, San Jose, CA, USA). During anesthesia at 5-min intervals the BIS values, heart rate (HR), systolic blood pressure (SBP), mean arterial blood pressure (MAP), diastolic blood pressure (DAP). For the infusion of ketofol and lidoketofol a Draeger™ Module DPS syringe pump (Draeger Medical Systems, Inc., Denver, MA, USA) was used.

### 2.3. Study Design

The study was performed as a randomized, prospective, double-blind clinical trial. The patients were randomly allocated into two groups, via a computer-generated randomization list. Both the patients and anesthesiologists were blinded to the drugs used. The anesthesiologist, who was blinded to the study’s medications, because of the medications prepared by a separate assistant who was not included into the study, administered the medications and evaluated the parameters. The medication that was prepared was the same color and shape. In one syringe ketamine–propofol (ketofol) combinations (esketamin, Ketanest, Pfizer, NY, USA) were prepared in one syringe, while the ketofol and lidocaine (Lidokain, Belupo, Koprivnica, Croatia) mixture was prepared in another syringe. The reduced manual infusion dose regimen for propofol (Propofol, Fresenius Kabi, Toronto, ON, Canada). The reduced McFarlan propofol infusion regimen was used for this study [11]. The infusion ratio was reduced to a value of BIS (65–70) [12,13].

Anesthetic monitoring such as non-invasive blood pressure, an ECG and a pulse oximeter were applied on the patients in the operating theater. The children were not premedicated and had been fasting for 6 h. If we did not have a peripheral intravenous (IV) cannula, anesthesia was then induced by means of sevoflurane (Sevorane, AbbVie, Campoverde di Aprilia, Italy) at 5–6% via a face mask. Ensuring a sufficient depth of anesthesia, the nurse then inserted a peripheral IV cannula. After the following, sevoflurane was then discontinued, and by intravenous means the induction drugs were administered. After providing intravenous access, the infusion of 0.9% NaCl solution or glucosaline infusion was set up and began. General anesthesia was then induced by ketofol or lidoketofol and fentanyl (Fentanyl, Piramal Critical Care Deutschland GmbH, Hallbergmoos, Germany), after 20 s, the laryngeal mask airway (LMA) was inserted, using a rotation technique with a partially inflated cuff [14,15]. The end-tidal carbon dioxide (ET-CO_2_) was maintained between 35 and 45 mmHg by mechanical ventilation. The maintenance of anesthesia was performed using infusion of ketofol or lidoketofol and using air/oxygen (50%/50%). The ketofol mixture was prepared in a ratio of 1:4 for induction and for maintenance 1:7. No lidocaine was added in the mixture of ketofol in group I. In group II, 20 mg of lidocaine was added in the mixtures of ketofol, for induction and the maintenance of anesthesia. Induction was performed with 3 mL per kg of mixture in both groups, and maintenance was provided with 80% or more of the McFarlan regimen in both groups. Tachycardia was defined as a 20% increase in HR and hypertension was defined as a 20% increase in MAP. If either or both HR and SBP increased to 20% of baseline, patients were given 0.5 μg/kg fentanyl.

For postoperative analgesia paracetamol in dose 10 mg/kg (Perfalgan, Bristol-Myers Squibb Pharmaceuticals limited, Bristol, UK) was administered to all patients. No drug was used as an antiemetic for postoperative nausea and vomiting (PONV). Ketofol or lidoketofol infusion had been discontinued, at the end of surgical operation. The time between ceasing the infusion and extubation is defined as the extubating time. When spontaneous regular breathing was confirmed the LMA was removed.

After the emergence from anesthesia children were monitored in the Post-Anesthesia Care Unit (PACU), where respiratory, heart rate and peripheral oxygen saturation (SpO_2_) were recorded and monitored. The time between arrival in PACU and discharge was described as the length of stay in PACU. The patients were discharged from PACU when a modified Aldrete score of ≥9 was achieved [16]. By continuous observations of the PACU nurses at 15 min intervals modified Aldrete scores were assessed in each patient. The patients were monitored for during the 24 h postoperatively and were closely observed for propofol infusion syndrome (PRIS).

### 2.4. Outcomes of the Study

The primary outcome of this study was the extubation time. Secondary outcomes were the time spent in the PACU, fentanyl and propofol consumption per kilogram (kg) and duration of anesthesia.

### 2.5. Sample Size Calculation

Sample size calculation has been decided according to the number of children that underwent elective short-term surgery (inguinal–scrotal region), in 2019, in our hospital. The calculation showed that minimum sample size required 192 patients for 95% confidence level and 5% margin of error. A total number of 200 patients (100 per group) were included to assure adequate power of the study and to prevent possible loss of the patients during the study period. Randomization was carried out by random blocks (*n* = 4).

### 2.6. Statistical Analysis

For data analysis the SPSS 24.0 software (IBM Corp, Armonk, NY, USA) was used. Median and interquartile range (IQR) were used to describe quantitative variables or an ordinal variable. Absolute and relative frequencies were used to describe quantitative variables or an ordinal variable. The significance of differences in quantitative variables between the study groups were evaluated by Mann–Whitney U test. The chi-square test was used to assess differences in distribution of categorical data. All the tests were two-sided and the significance level of 0.05 was used.

## 3. Results

A total of 200 patients were eligible for the study. No significant differences in regards to demographic data, hemodynamic data (DAP, MAP, HR), SpO_2_ and BIS values were found between the investigated groups. Mean BIS values were around 65–70 for both groups. Demographic data of the patients are shown in Table 1 while the comparison of variables investigating main outcomes of the study is shown in Table 2.

**Figure 2:**
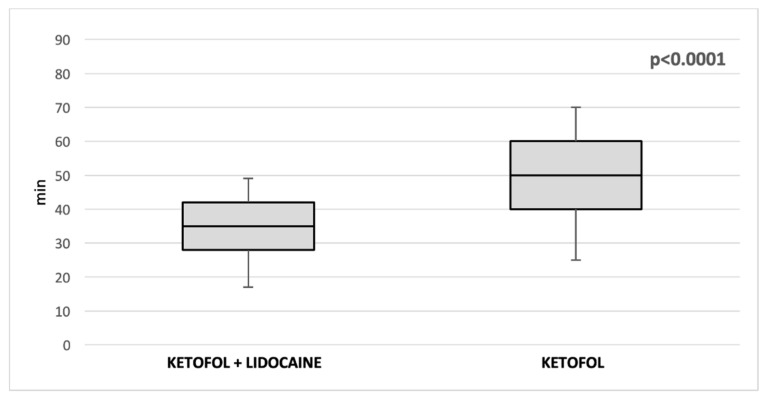
Comparison of median duration of anesthesia between two groups.

**Figure 3:**
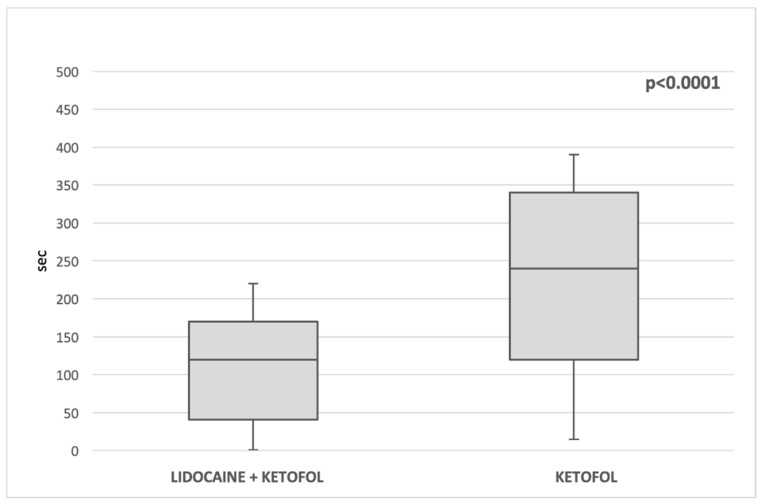
Comparison of median extubating times between two groups.

No patient had hemodynamic values exceeding 20% of pre-anesthetic values. Anesthesia duration (35 min (IQR 28, 42) vs. 50 min (IQR 40, 60); *p* < 0.00001) (Figure 2) and extubating times were significantly shorter (120 s (IQR 41.5, 170) vs. 240 s (IQR 120, 340); *p* < 0.00001) (Figure 3) in lidoketofol group. In the lidoketofol group the consumption of fentanyl (2.1 µg/kg (IQR 2, 2.8) vs. 2.3 µg/kg (IQR 2, 3); *p* < 0.0056) and propofol (6.6 mg/kg (IQR 4, 7.5) vs. 7.6 mg (IQR 5, 10.5; *p* < 0.032) per kg were significantly lower.

**Figure 4:**
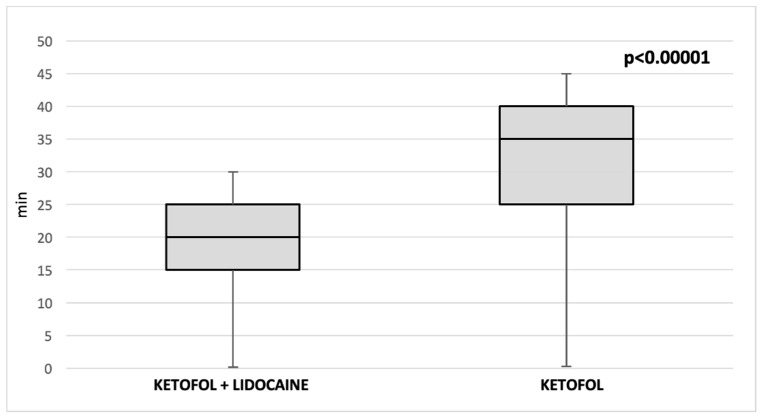
Comparison of median length of stay in the PACU between two groups.

Length of stay in the PACU was significantly shorter (*p* < 0.00001) in the ketofol and lidocaine group (20 min; IQR 20, 25) than in the ketofol group (35 min; IQR 30, 40) (Figure 4). Vomiting was observed in one patient in each group. One patient out of both groups presented with signs of mild laryngospasm. In no way did any of the patients show signs of propofol-related infusion syndrome (PRIS).

## 4. Discussion

This study is the first to compare ketofol and ketofol with lidocaine for induction and maintenance of anesthesia. TIVA with ketofol, using the reduced McFarlan regimen, is effective in pediatric population especially during short surgical procedures. The present study suggests that TIVA with lidoketofol fusion, with an 80% or more reduced McFarlan regimen can ensure a comfortable perioperative and postoperative recovery. However, this study reveals the lidoketofol group has shown a statistically significant shorter extubating time and shorter postoperative time spent in the PACU. The consumption of fentanyl and propofol per kg was lower in the lidoketofol group. We should also mention that hemodynamic stability was provided by lidoketofol infusions such as adequate postoperative analgesia and successful recovery for short operative pediatric procedures.

TIVA has become a favored option for pediatric anesthesia during the past years; TIVA is considered an alternative option in pediatric general anesthesia according to some previous studies. Many advantages of the use of TIVA during pediatric general anesthesia were stated by Lauder, such as a reduction in laryngospasm, airway reactivity, postoperative nausea and vomiting, emergence delirium, postoperative pain, and a reduction of stress hormones [17]. For TIVA application in pediatric population propofol is commonly used. Improved hemodynamic conditions are ensured by the addition of ketamine as an adjuvant agent to propofol. Furthermore, the addition of ketamine provides greater postoperative analgesia and a greater emotional state [7]. Dallimore et al. investigated an infusion regimen with ketamine that was able to preserve a steady blood concentration [18]. The pharmacodynamics properties produced by ketamine are similar in children (apart from infants) and in adults [19]. Biricik et al. compared different combinations of ketofol in pediatric population and revealed that TIVA with a ketofol ratio of 1:10 can contribute to improved recovery conditions [20]. An elevation in BIS values can occur due to ketamine, approximately 65–70 [12,13]. Due to the inclusion of ketamine the degree of anesthesia is difficult to evaluate. Coulter et al. presented the ketofol dosing simulation in pediatric population for anesthesia [21]. A 1:5 ratio of ketamine–propofol mixture for anesthesia of a duration of 30 min and a 1:6.7 ratio of ketamine–propofol for 90 min anesthesia are proposed to be the best choice for ketofol infusion. This simulation suggests that ratios of ketofol such as 1:5, 1:6.7, and 1:10 can be practical for short-term anesthesia without lengthening the recovery. Accordingly, our ratio of ketofol were 1:4 for induction and 1:7 for maintenance. Short duration surgical operations with the maximum of 60 min, were cases we aimed to include into our study.

In numerous studies, after the lidocaine infusion was stopped a persistent analgesic effect was present. Perioperative lidocaine has a protective effect on postoperative pain for up to 72 h following abdominal surgery [22]. A randomized, double-blind, placebo-controlled study in which 36 adult patients underwent breast cancer surgery exhibited a decrease in the incidence and intensity of chronic pain following the procedures, with the use of perioperative intravenous lidocaine (bolus of IV lidocaine 1.5 mg/kg followed by continuous infusion of lidocaine at 1.5 mg/kg/h). It is well-known that application of lidocaine at the site of surgical incision reduces postoperative pain [23], but clinical evaluations suggested that the use of intravenous lidocaine therapy in this way has beneficial effects on pediatric postoperative pain, the opioid demand and the sense of well-being of children, especially during the 24 h postoperatively [24]. Sun et al. published a meta-analysis of randomized controlled trials where they explored the use of systemic lidocaine for postoperative analgesia and recovery after abdominal surgical procedures [25]. A decrease in the intensity of postoperative pain, opioid use, time to first bowel movement and length of hospital stay was shown to occur. In a retrospective analysis of data, in a limited number of pediatric patients undergoing general anesthesia for laparoscopic appendectomy, continuous perioperative administration of intravenous lidocaine did not produce any adverse effects [26]. Consistent with our study, Fang et al. supplied information supporting the use of the ketamine and lidocaine mixture which contributed to more stable vital signs, shorter onset and recovery time, elimination half-life prolonged, increased area under the curve, decreased plasma clearance, dosage and adverse effects [27]. Our results have shown that TIVA with lidoketofol decreased opioid consumption and shortened the extubating and recovery time.

The present study has several limitations. Firstly, no plasma concentrations of propofol, ketamine or lidocaine were measured. Secondly, no scale was used for detection of emergence agitation. Lastly, the authors assumed that due to similar hemodynamic and BIS values the anesthesia depth is equivalent between groups.

## 5. Conclusions

The results of this study clearly demonstrated that extubating time, as well as the anesthesia duration were significantly shorter when lidocaine was administrated together with ketofol, compared to administration of ketofol alone. Apart from the extubating time and length of stay in the PACU, fentanyl and propofol consumptions per kg were significantly lower in patients who received admixture of ketofol and lidocaine. Both groups showed similarities between the perioperative hemodynamic parameters and postoperative pain scores.

## Figures and Tables

**Table 1:** Demographic and clinical data of the patients.

	Group I	Group II	*p*
	KETOFOL	KETOFOL + LIDOCAINE	
Age (years) (median, IQR)	5	5	0.681 *
	(4, 7)	(3, 7)	
Sex (M) (*n*, %) (F) (*n*, %)	78 (78%)	81 (81%)	0.599 **
	22 (22%)	19 (19%)	
Weight (kg) (median, IQR)	21	10.5	0.841 *
	(16, 28)	(15, 32.5)	

* Mann–Whitney test, ** Chi-square test; IQR—Interquartile range; PACU—Post-anesthesia care unit.

**Table 2:** Comparison of main outcomes of the study between two investigated groups.

Variable	Group I	Group II	*p*
	KETOFOL	KETOFOL + LIDOCAINE	
Sevoran (iv cannula) (*n*, %)	64 (64%)	54 (54%)	0.150 **
Length of iv infusion (min) (median, IQR)	23 (16, 30.5)	23 (16, 30)	0.944 *
Duration of anesthesia (min) (median, IQR)	50 (40, 60)	35 (28, 42)	<0.00001 *
Extubation time (s) (median, IQR)	240 (120, 340)	120 (41.5, 170)	<0.00001 *
Fentanyl (μg/kg) (median, IQR)	2.3 (2, 3)	2.1 (2, 2.8)	<0.0056 *
Fentanyl—TOTAL (μg) (median, IQR)	50 (40, 60)	45 (30, 65)	0.250 *
Propofol (mg/kg) (median, IQR)	7.55 (5, 10.5)	6.6 (4, 7.5)	0.032 *
Propofol—TOTAL (mg) (median, IQR)	162.5 (122.5, 217.5)	135 (106.5, 204)	0.014 *
Ketamin (mg/kg) (median, IQR)	1.6 (1.2, 2.1)	1.3 (1, 1.5)	<0.0001 *
Ketamin—TOTAL (mg) (median, IQR)	32 (24, 36)	29 (22, 32)	0.378
PACU (min) (median, IRQ)	35 (30, 40)	20 (20, 25)	<0.00001 *

* Mann–Whitney test, ** Chi-square test. IQR—Interquartile range; PACU—Post-anesthesia care unit.

## Data Availability

The data presented in this study are available upon request of the respective author. Due to the protection of personal data, the data are not publicly available.
